# Impact of Dental Procedures on Hereditary Angioedema Attacks: An Exploratory Observational Study 

**DOI:** 10.3290/j.ohpd.c_1907

**Published:** 2025-03-14

**Authors:** Valentin Nadasan, Konrád-Ottó Kiss, Réka Borka-Balás, Noémi-Anna Bara

**Affiliations:** a Valentin Nadasan Professor, Department of Hygiene, George Emil Palade University of Medicine, Pharmacy, Science, and Technology of Targu Mures, Romania. Study design and methodology, data acquisition, analysis and interpretation, drafting original draft, revision and approval of the final manuscript.; b Konrád-Ottó Kiss Medical Doctor, Mures Emergency Clinical County Hospital, Targu Mures, Romania. Data acquisition, revision of the manuscript, approval of the final version of the manuscript.; c Réka Borka-Balás Lecturer, 1st Department of Pediatrics, George Emil Palade University of Medicine, Pharmacy, Science, and Technology of Targu Mures, Romania. Study design, drafted original draft, reviewed and approved final manuscript.; d Noémi-Anna Bara Medical Doctor, Doctoral School, George Emil Palade University of Medicine, Pharmacy, Science, and Technology of Targu Mures, Romania; Hereditary Angioedema Expertise Centre, Sangeorgiu de Mures, Romania. Study design and methodology, data acquisition, analysis and interpretation, drafted original manuscript, revised and approved the final manuscript.

**Keywords:** dental care for the chronically ill, disease management, health services accessibility, hereditary angioedema, orphan diseases

## Abstract

**Purpose:**

To evaluate hereditary angioedema (HAE) attack frequency associated with dental procedures, determine whether patients with post-dental procedural attacks receive more appropriate treatment after their condition is diagnosed, and investigate the potential impact of perceived risk on patients seeking dental care and dental professionals providing it.

**Materials and Methods:**

The observational study included all the eligible adults from the Romanian Hereditary Angioedema Registry who provided informed consent. The impact of dental procedures on the HAE attacks was measured using a structured questionnaire including 20 questions administered via telephone.

**Results:**

Patients experienced dental procedure-related symptoms suggestive of HAE both before (47.6%) and after their condition was diagnosed (51.9%). Before the HAE diagnosis, 86.2% of the patients received glucocorticoids and antihistamines for post-procedural swelling. After diagnosis, 85.3% of the patients were given Icatibant and C1-INH. More than half (55.3%) of the patients reported not seeking dental interventions because of fear of HAE attacks or anticipation of refusal, and 24.7% of them declared they had been denied dental care by dental health professionals at least once.

**Conclusion:**

Swelling related to dental procedures was common among the studied HAE patients. Unwarranted medications used before HAE diagnosis for dental post-procedural symptoms were replaced by adequate HAE-specific medications in most patients with established HAE diagnosis. A statistically significant proportion of patients refrained from undergoing dental interventions, and some of them were refused dental care by oral health professionals due to fear of HAE attacks.

Hereditary angioedema (HAE) is a rare, life-threatening condition of genetic etiology. HAE related to C1-esterase inhibitor deficiency (C1-INH) is caused by mutations on the SERPING1 gene and has an estimated prevalence of 1:50,000 in the general population.^1,24^ HAE is classified into type I HAE, characterised by deficient levels and function of the C1-INH protein; type II HAE, characterised by expected levels but dysfunctional C1-INH protein; and type III HAE, characterised by normal levels and function of C1-INH, currently referred to as HAE with normal C1-INH.^6^ The clinical manifestations include subcutaneous and/or submucosal edema of the limbs, face, digestive tract, and upper respiratory airways, but medications such as antihistamines, glucocorticoids, and epinephrine are not effective.^7^ Unless promptly treated, laryngeal HAE attacks may lead to death by asphyxia.^4^ Known factors causing swelling are mechanical trauma, emotional stress, surgical or diagnostic procedures performed in the head and neck area, infections, hormonal changes (puberty, menstruation, pregnancy), and certain drugs (oral contraceptives, angiotensin-converting enzyme inhibitors).^10,21^

Dental operations, such as tooth extractions or even minor manipulations like obtaining dental impressions or pulpal excavation, are one of the main causes of angioedema attacks with possibly critical consequences. The swelling may manifest as late as 24 to 48 hours after the dental procedure.^11,23^ In the absence of prophylactic medication, the risk of perioperative angioedema was estimated between 5.7% and 30.5%.^2^ HAE patients face challenges accessing dental care, leading to poor oral health.^25^ Even when dental services are covered by insurance, HAE patients with a history of post-dental procedure attacks show reduced demand for dental services compared to the general population.^22^ HAE patients experience higher levels of anxiety compared to the general population. Anxiety has been linked with oral surgery distress, augmented pain perception, and overall lower quality of life. Studies suggest an association of anxiety with the unpredictability, severity, social or professional impact of the HAE attacks, and the risk of passing the disease to the offspring.^8,20^ HAE specialists widely concur that anxiety may worsen the course of the acute phase of the disease or even trigger HAE attacks, leading to a self-aggravating spiral.^15,20^

Current HAE management guidelines recommend short-term prophylaxis (STP) before dental procedures, especially when more traumatic interventions are performed. Routine prophylaxis is not necessary for milder manipulations, but on-demand medication should be available without delay.^3,4^ STP may consist of plasma-derived C1-INH (pdC1-INH) administered in a single dose as close as possible to the start of the procedure.^6,16^ When unavailable, recombinant human C1-INH (rhC1-INH) or fresh frozen plasma (FFP) should be administered. Attenuated androgens have been administered in the past for STP 5 days before and 2–3 days post-event.^6,18^ Routine prophylactic treatment with C1-INH improves overall quality of life, including parameters related to anxiety.^17^ Preprocedural anxiety assessment, especially in patients with intense odontophobia possibly followed by pharmacological sedation, preferably through oral or nasal routes, may be essential in the management of HAE patients.^20^

Although post-dental–procedure HAE attacks represent a concern for both patients and dental professionals, there is no data about this issue in Romanian HAE patients, and evidence from other countries is minimal. This study aimed to evaluate HAE attack frequency related to dental procedures, determine if post-procedural attacks receive more appropriate treatment after diagnosis, and investigate whether the perceived risk of dental procedure-related angioedema hinders patients from seeking dental care and discourages dental professionals from providing it.

## Materials and Methods

The research was designed as an observational study. The study was approved by the ethics committee and the IRB of the George Emil Palade University of Medicine, Pharmacy, Science, and Technology of Targu Mures, Romania (Decision no. 1306/19.03.2021).

All eligible adults enrolled in the Romanian Hereditary Angioedema Registry were invited to participate. Patients were screened for eligibility based on inclusion (patients with laboratory-confirmed diagnosis of hereditary angioedema; male or female patients 18 years of age; patients with permanent residency in Romania; patients providing informed consent) and exclusion criteria (male or female patients under 18 years of age; patients with current symptoms of psychosis or dementia; patients with severe hearing impairment; patients not able to provide informed consent).

Information regarding patient eligibility (age, confirmed diagnosis of HAE and comorbidities preventing patients’ participation in the study) and type of HAE were collected from the Romanian Hereditary Angioedema Registry.

The impact of dental procedures on the HAE attacks was measured using a structured questionnaire including 20 items developed specifically by the authors of this study, as there were no existing validated tools for this purpose. The first author prepared the questionnaire draft considering the previously agreed objectives of the study. Next, an HAE expert reviewed the draft independently for relevance and appropriateness of the items, language, and structure. Then, the observations were integrated into the second version, which was reviewed in a group meeting by all authors. The main questions addressed in this survey were dental services utilisation before and after HAE diagnosis, the occurrence rate of attacks following dental procedures, the type and frequency of medications administered after these procedures, the number of instances when patients were denied dental care by oral health professionals or the patients refrained from dental interventions due to fear of HAE attacks or anticipation of refusal. The complete questionnaire is available in the Appendix.

**Fig 1:**
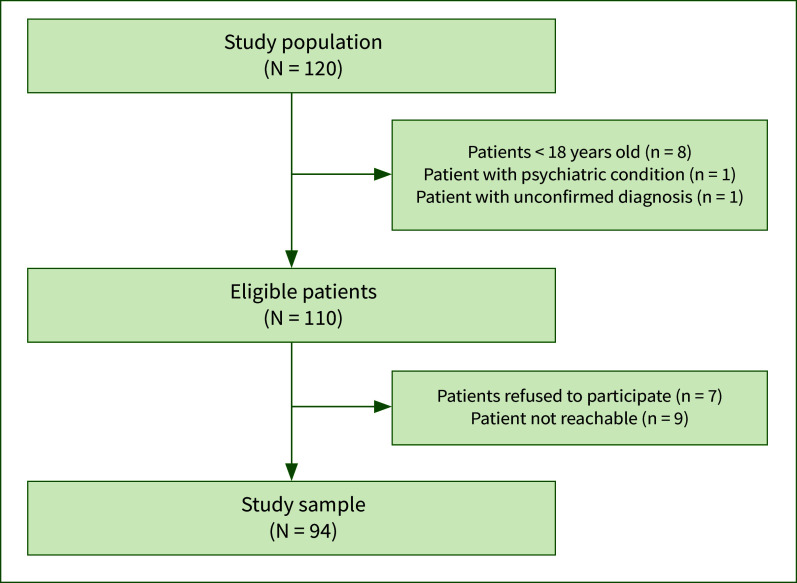
Flow diagram of the study.

During the initial phone call, patients were informed about the purpose of the study, the procedures, and their rights as participants. Patients who provided verbal informed consent were scheduled for a subsequent session during which trained personnel administered the questionnaire via telephone, following specific instructions. The size of the study population, the number of eligible, lost and responding patients are presented in Fig 1.

Absolute and relative frequencies of the categorical variables, the mean values, and the standard deviations of the numerical variables were computed. Socio-demographic characteristics of respondent vs non-respondent HAE patients were compared using the chi-squared or Fisher’s exact test, Student’s t-test or Mann-Whitney test as appropriate. Comparisons between variables before and after diagnosis were performed using the McNemar test for questions with binary answers and the McNemar-Bowker test for questions with three-level answers. The statistical significance threshold was set at α = 0.05.

## Results

A total of 94 patients participated in the study (response rate 78.3%). The sociodemographic and disease-related characteristics of the patients who were/were not included in the study are presented in Table 1.

**Table 1:** The sociodemographic and disease-related characteristics of respondent and non-respondent HAE patients

Variable	Respondents (n = 94)	Non-respondents (n = 16)	p-value
Age, years: mean (SD)	45.3 (14.3)	43.4 (13.9)	0.623^†^
Sex: n (%)
Female	62 (66.0)	8 (50.0)	0.220^‡^
Male	32 (34.0)	8 (50.0)	
Residence: n (%)
Urban	60 (63.8)	11 (68.8)	0.704^‡^
Rural	34 (36.2)	5 (31.2)	
HAE type: n (%)
HAE type I	83 (88.3)	5 (93.8)	1^§^
HAE type II or III	11 (11.7)	1 (6.2)	
Age at first symptom, years: mean (SD)	15.6 (11.8)	21.1 (14.8)	0.130^¶^
Age at diagnosis, years: mean (SD)	36.0 (14.6)	39.0 (15.0)	0.446^‡^
Diagnosis delay, years: mean (SD)	20.3 (14.2)	17.9 (13.5)	0.552^¶^
^†^p-value of t-test for equality of means, independent samples; ^‡^p-value of chi-squared test; ^§^p-value of Fisher’s exact test; ^¶^p-value of Mann-Whitney test.

During the last 6 months, all 94 patients included in the study had access through the National HAE Program to home treatment with subcutaneous injection of bradykinin B2-receptor antagonist for acute HAE attacks (icatibant), and 7 (7.4%) had also used pdC1-INH. STP, consisting of pdC1-INH, was used by 11 patients (11.7%), while 1 patient (1.1%) reported receiving FFP for the same purpose. Long-term prophylaxis (LTP) consisting of pdC1-INH was administered to 8 (8.5%) and danazol was given to 3 patients (3.2%). pdC1-INH was available only during the last month of the study period. Additionally, 6 patients (6.4%) received HAE-specific medication in the emergency department (2 patients: rhC1-INH; 2 patients: pdC1-INH; 2 patients: FFP).

Patients’ responses to questions regarding the use of dental services, observed post-procedural attacks, and medications administered in case of such events, before and after being diagnosed with HAE, are reported in Table 2.

**Table 2:** Descriptive statistics of dental services use, post-procedural attacks, and medications received by patients, before and after being diagnosed with hereditary angioedema

Questions/Answers	Before HAE diagnosis n (%)	After HAE diagnosisn (%)	Pre- vs post-diagnosis comparison test p-value
Have you ever seen a dentist?	N = 94	N = 94	>0.99^†^
Yes	82 (87.2)	81 (86.2)
No	12 (12.8)	13 (13.8)
If yes, how often?	N = 82	N = 81	0.916^‡,§^
Less than once a year	35 (42.7)	33 (40.7)
Once or twice a year	36 (43.9)	36 (44.4)
More than twice a year	11 (13.4)	12 (14.8)
Have you ever had any swelling or a feeling of suffocation after a dental procedure?	N = 82	N = 81	0.824^†,§^
Yes	39 (47.6)	39 (51.9)
No	43 (52.4)	42 (48.1)
If yes, how many times?	N = 39	N = 39	0.0292^‡,¶^
1–5 times	18 (46.2)	21 (53.8)
6–10 times	8 (20.5)	5 (12.9)
More than 10 times	13 (33.3)	13 (33.3)
Have you ever been given any treatment for the swelling/feeling of suffocation you experienced after the dental procedure?	N = 39	N = 39	0.219^†,¶^
Yes	29 (73.0)	34 (87.2)
No	10 (27.0)	5 (12.8)
If yes, how many times?	N = 29	N = 34	0.675^‡,#^
1–5 times	19 (65.5)	19 (55.9)
6–10 times	2 (6.9)	4 (11.8)
More than 10 times	8 (27.6)	11 (32.3)
Please specify what treatment you were given	N = 29	N = 34	
Glucocorticoids and/or antihistamines	25 (86.2)	9 (26.5)	0.006^‡,#^
Icatibant, C1-INH, FFP, and/or danazol	2 (6.9)	29 (85.3)	<0.001^†,#^
Analgesics, NSAIDs, and/or other medication	8 (27.6)	7 (20.6)	0.219^†,#^
How did you feel after the treatment?	N = 29	N = 34	0.004^‡,#^
Better	6 (20.8)	26 (76.5)
The same	21 (72.4)	2 (5.9)
Sometimes better, other times worse or the same as before the treatment	2 (6.9)	6 (17.6)
HAE: hereditary angioedema; ^†^McNemar test; ^‡^McNemar-Bowker test; ^§^72 respondents with complete data; ^¶^25 respondents with complete data; ^#^ 18respondents with complete data.

Patients’ responses to questions regarding the administration of pre-procedural medications to prevent HAE attacks are presented in Table 3.

**Table 3:** Frequency, type, and perceived efficacy of pre-procedural medication administered to hereditary angioedema patients in the dental setting

Questions/Answers	n (%)
Have you ever been given any medication to prevent the attack of hereditary angioedema (before a dental procedure)?	N = 75
Never	53 (70.7)
Every time	6 (8.0)
Sometimes	16 (21.3)
Please specify the preventive medication you were given.	N = 22
Danazol	10 (45.5)
pdC1-INH	6 (27.3)
FFP	3 (13.6)
rhC1-INH	3 (13.6)
Icatibant	2 (9.1)
Epinephrine	1 (4.5)
Other	2 (9.1)
Have you ever had swelling or a feeling of suffocation after a dental procedure performed under preventive medication?	N (22)
No	6 (27.3)
Yes, but they were less severe	12 (54.5)
Yes, and they were as severe as when you did not receive preventive medication	4 (18.2)


Patients’ answers to questions assessing the frequency of situations when they were denied or did not ask for dental services are reported in Table 4.

**Table 4:** Assessment of the frequency of situations when hereditary angioedema patients were denied or did not ask for dental services

Questions/Answers	n (%)
Has it ever happened to you that the dentist refused to perform a dental procedure on you because of hereditary angioedema?	N = 81
Yes	20 (24.7)
No	61 (75.3)
If yes, how many times?	N = 20
1–2 time	10 (50.0)
3–5 times	4 (20.0)
6–10 times	3 (15.0)
More than 10 times	3 (15.0)
Have you ever needed dental treatment but still did not see a dentist?	N = 94
Yes	42 (55.3)
No	52 (44.7)
Please state the reasons why (you may choose more than one answer).	N = 42
Fear of an attack	33 (78.6)
Pain or discomfort caused by the procedure	8 (19.9)
Fear of being refused by the dental professional	4 (9.5)
Other reasons	5 (11.9)


The data that support the findings of this study are available from the corresponding author upon reasonable request.

## Discussion

Almost half of the HAE patients in the study sample experienced dental procedure-related symptoms suggestive of HAE both before and after their condition was diagnosed. After the HAE diagnosis, the pattern of post-procedural swelling treatment underwent a major shift from inefficient medications such as glucocorticoids and antihistamines to HAE-specific medications. Preventive medication for dental intervention-related HAE attacks was consistently administered to only a small minority of respondents. Finally, the study observed a considerable proportion of participants who were denied dental care by dental health professionals, while half of the patients acknowledged they did not demand dental interventions because of fear of HAE attacks or because of anticipation of refusal.

The investigation found that a large majority of the responding patients had seen a dentist both before and after being diagnosed with HAE (87.2% and 86.2%, respectively), and there was no statistically significant difference between the two periods. The majority of those seeing a dentist did so once or more than once a year; being diagnosed with HAE did not change the frequency of dental visits (Table 2). Although a rigorous comparison is not possible, the proportion of people visiting a dental professional at least once a year seems very low among HAE patients (50.5% when those not seeing a dentist at all were included in the calculation) compared to the general population from Romania (71%).^9^ A decrease in routine dental visits in patients experiencing repeated HAE attacks subsequent to dental interventions was also reported by Singh et al.^22^ Diminished access to dental services may be related to the external and internal obstacles HAE patients face when needing dental care. Almost a fourth of the patients diagnosed with HAE in our sample (24.7%) reported being denied a dental procedure at least once because of the disease. Half of the patients (50.0%) remembered being denied dental procedures just one or two times, while some of them declared their request was declined more often (in a few cases even more than 10 times). Moreover, a little over half of the respondents (55.3%) acknowledged that they did not seek a dental professional despite the need for professional help. While fear of an attack was the most common reason for not demanding dental care (78.6% of the patients), in a few cases (9.5% of the patients; statistically significant), the reason was to avoid the embarrassment of being refused by the dental professional (Table 4). In a study on 29 HAE patients from Italy seeking consultation in the dentistry department, 63.0% reported having difficulties in securing the needed care (caries, tooth extraction, etc).^25^ In an earlier study by Lodi et al,^14^ which included 57 HAE patients, 19 experienced some challenges in obtaining dental assistance and one patient was refused care.^14^ The barriers the patients encounter may be connected to the low awareness and knowledge regarding the management of this rare disease among dental professionals.^25^

The overall proportion of patients reporting dental procedure-related symptoms suggestive of HAE attacks was around half, both before (47.6%) and after (51.9%) the timepoint of HAE diagnosis. However, a small but statistically significant improvement was observed in our study when looking at the changes in the frequency of HAE attacks following dental interventions. The number of patients with 6 to 10 post-procedural attacks decreased by almost 10% after the diagnosis, while the subcategory of patients reporting 1 to 5 attacks increased by the same percent (Table 2). These trends may be reasonably attributed to an overall improved access to HAE-specific medication, particularly STP and LTP.

When medications administered for post-procedural attacks were considered indiscriminately, neither the number of patients nor the frequency of receiving medications was statistically significantly different before and after diagnosis, respectively. However, the study revealed substantial changes in the medication given to patients with established HAE diagnosis. The administration of glucocorticoids (e.g., hydrocortisone, prednisone) and antihistamines after the diagnosis decreased more than three times (86.2% before diagnosis vs. 26.5% after diagnosis), while HAE-specific medication (icatibant, C1-INH, FFP) and danazol were given 12 times more often (6.9% before diagnosis vs. 85.3% after diagnosis). This massive shift in the pattern of pharmacological intervention in response to dental post-procedural HAE attacks probably reflects the positive impact of the National HAE Program ensuring free access to HAE-specific medication to all registered patients. Additionally, the educational activities delivered by the HAE Expertise Center to patients, treating physicians, and emergency department personnel may also have contributed to these favourable changes. These transformations are all the more impressive considering that icatibant and pdC1-INH became available through the National Program only in 2017 and 2021, respectively.^12^

Overall progress in the management of HAE in Romania is also suggested by the higher proportion of respondents who reported feeling better after medication for dental-treatment–related symptoms was administered. More precisely, the proportion of respondents feeling better after receiving post-procedural medication was almost four times higher among patients with HAE diagnosis compared to those not yet diagnosed (29.8% before diagnosis vs 76.5% after diagnosis). Accordingly, the proportion of respondents declaring that medication did not affect their post-procedural symptoms decreased more than 12 times after HAE diagnosis (72.3% before diagnosis vs 5.9 after diagnosis). The non-negligible proportion of patients (20.6%) receiving analgesics and non-steroidal anti-inflammatory drugs even after diagnosis may simply represent the common usage of such medication in dental practice.^19^

The findings regarding preventive pre-procedural drugs also point out some matters of concern. Very few patients were given such medication every time a dental intervention was performed (8.0%). In addition, about a fifth of the patients (21.3%) received this type of medication only sometimes, while the large majority (70.7%) have never been administered pharmacological prevention of dental-procedure–related attacks. These figures seem to resemble the findings of Bork et al^5^ in a group of 171 HAE patients in Germany, with 123 patients (71.9%) who underwent tooth extractions never receiving any preventive medicine. Given that pharmacological prophylaxis has become available through the HAE National Program as late as 2021, the low number of Romanian HAE patients benefiting of such medicines is not unexpected. When preventive medication was offered, danazol was the most common (almost half of the interventions), followed by pdC1-INH and FFP. The predominant use of attenuated androgens in Romania is very likely due to their lower cost. Current guidelines emphasize the importance of STP in dental surgery, which is associated with swelling of the oral and perioral region that can cause life-threatening laryngeal edema. STP of such events may consist of a single dose of pdC1-INH or a course of attenuated androgens.^3,6^

More than a fourth of the patients did not report post-procedural attacks when the interventions were performed under prophylactic medication, while more than half reported less severe attacks. Less than one in five declared that the attacks were just as severe as when not receiving preventive medication before the dental procedure. These observations are in line with data published by other authors who reported either the absence or substantial reduction of post-procedural HAE attacks in patients receiving STP.^5,13,25^ Finally, these data further emphasise the potential benefits of improving the implementation of the National HAE Program to cover the HAE patients’ needs in the dental setting.

As a practical implication, the study suggests that HAE management in Romania could benefit from educational efforts to increase awareness and knowledge regarding HAE, especially post-procedural attack prevention among dental practitioners. HAE experts recommend that dental professionals should be informed about this rare condition and trained to proactively include a specific question in the anamnesis regarding recurrent subcutaneous or submucosal edema suggestive of HAE attacks.^11^

### Strengths and Limitations

This is the first study exploring the impact of dental procedures on post-procedural swellings in HAE patients from Romania. The sample included 94 of the 110 eligible patients listed in the HAE Registry and had a very good response rate (85.5%), even considering the small sample size. Furthermore, non-response bias may be ruled out based on the comparative analyses of respondent vs non-respondent patients (Table 1), which showed no statistically significant differences regarding basic sociodemographic and disease-related characteristics. Therefore, the results may safely be extrapolated to the adult registered HAE population. To the best of our knowledge, this is the only study to document differences in medication administered for dental procedure-related HAE attacks in relation to HAE diagnostic status.

The potential limitations are related to respondent biases, data collection methods, and the number of respondents answering some questions. First, and most important, the questionnaire used to collect the data did not undergo a complete formal process of validation due to time, resource, and sample size constrains. The study’s reliance on such an instrument may affect the reliability and validity of its findings. Its ability to consistently and accurately capture the intended constructs, such as the impact of dental procedures on HAE attacks or barriers to care, remains uncertain. This limitation may also raise concerns about response variability, measurement errors, and difficulties in benchmarking results against other studies. The authors exercised due diligence to develop the best possible instrument for the survey, but future studies using a thoroughly validated questionnaire to confirm the results are warranted. Second, as expected, recall bias may have affected the accuracy of responses, as the patients were asked about symptoms and medications related to events that happened months or even years ago. In order to mitigate this undesired effect, the operator was specifically instructed to assure the respondents that they could take as much time as needed to remember the facts under investigation. Third, to address social desirability bias, which is often present in face-to-face or telephone interviews conducted by individuals known to the respondent, the study ensured that participants’ answers would be anonymous and only disclosed in aggregate form. Additionally, an interviewer unknown to the patients was employed to administer the questionnaire. Finally, as the data collection tool included a series of conditional questions, the number of patients answering the questions was progressively smaller therefore the statistical analyses of these data may be less robust compared to data based on the very first questions. Implementing such a hierarchical approach was necessary to ensure that the participants were asked only the relevant questions and to minimise response errors.

## Conclusion 

Nearly half of the HAE patients in the sample experienced symptoms related to dental procedures before and after their condition was diagnosed. Ineffective medications used by most patients before HAE diagnosis were replaced by HAE-specific, effective medication in the majority of the patients with established HAE diagnosis. Finally, a statistically significant proportion of participants were denied dental care by dental health professionals and half of the patients did not request dental interventions due to fear of HAE attacks or anticipation of refusal.

## Conflict of interest

VN and NB are members of the Romanian Registry for HAE Foundation and have received financial support for this research, lecturing and travel from Takeda Pharmaceuticals.

We thank Dragoș Chetroiu, Claudiu Pintilie, Anamaria Barbu from Takeda Pharmaceuticals S.R.L for their assistance in study oversight. We thank Nicoleta Radu and Attila Borka-Balás from the Romanian Registry for HAE Foundation for administrative support. This study was financially supported by Takeda Pharmaceuticals S.R.L. The company ensured study oversight, contributed with clarification questions but had no involvement in editorial decisions regarding interpretation of the study results or restrictions regarding publication.

## References 
